# Bovine Immunology: Implications for Dairy Cattle

**DOI:** 10.3389/fimmu.2021.643206

**Published:** 2021-06-29

**Authors:** Anastasia N. Vlasova, Linda J. Saif

**Affiliations:** Center for Food Animal Health, Ohio Agricultural Research and Development Center, Department of Animal Sciences, College of Food, Agricultural and Environmental Sciences, The Ohio State University, Wooster, OH, United States

**Keywords:** bovine, immune responses, infectious diseases, dairy cows, neonatal calves, cattle

## Abstract

The growing world population (7.8 billion) exerts an increased pressure on the cattle industry amongst others. Intensification and expansion of milk and beef production inevitably leads to increased risk of infectious disease spread and exacerbation. This indicates that improved understanding of cattle immune function is needed to provide optimal tools to combat the existing and future pathogens and improve food security. While dairy and beef cattle production is easily the world’s most important agricultural industry, there are few current comprehensive reviews of bovine immunobiology. High-yielding dairy cattle and their calves are more vulnerable to various diseases leading to shorter life expectancy and reduced environmental fitness. In this manuscript, we seek to fill this paucity of knowledge and provide an up-to-date overview of immune function in cattle emphasizing the unresolved challenges and most urgent needs in rearing dairy calves. We will also discuss how the combination of available preventative and treatment strategies and herd management practices can maintain optimal health in dairy cows during the transition (periparturient) period and in neonatal calves.

## Introduction

Early fetal and neonatal calf mortality is a major contributor to increased production costs. Also, during the transition period (~3 weeks prior to and ~3 weeks after calving), dairy cows experience immune and metabolic dysregulation, that makes them very vulnerable to various infectious and non-infectious diseases. Despite the widespread availability of vaccines and antimicrobial compounds, several infectious diseases continue causing substantial morbidity, mortality, and economic loss to the cattle industry. To maintain optimal health in a cattle herd it is critical to understand mechanisms of natural anti-infectious immunity and how vaccination, biosecurity, nutrition, husbandry and calf management practices should be used to maintain and enhance immune protection. The advent of new high-throughput sequencing technologies and the publication of the complete bovine genome (in 2009) have boosted research that significantly enhanced our knowledge of the immune response in cattle. While this novel knowledge is being used actively for breeding and selection of cattle with desired performance and health traits, methods to genetically improve infectious disease resistance do not exist yet (1).

Mycobacterium and mammary gland (mastitis) infections represent two major threats impacting global cattle production and reaching $35 billion in annual costs globally (2, 3). Respiratory infections are commonly associated with multiple pathogens and therefore referred to as bovine respiratory disease (BRD) complex (4). They are the principal source of significant economic losses for the North American beef and dairy industries. A number of bacteria and viruses are known to be associated with BRD in combination with other stress factors including heat, cold, fatigue, inadequate hydration or nutrition, injury or environmental dust contamination (4, 5). Similarly, several viral and bacterial pathogens are known to cause severe enteric diseases in calves and adult cattle. The major pathogens associated with scours are bovine coronavirus, bovine rotavirus, bovine viral diarrhea virus, salmonella spp. (multiple serotypes Dublin, Heidelberg, and Newport), *Escherichia coli* K99, cryptosporidia and *Clostridium perfringens* (6–8). With majority of vaccines against enteric pathogens having low efficacy or lacking broad protection, the threat of antimicrobial resistance (AMR) and ensuing decrease in the use of antimicrobials and often multi-agent nature of scours, maintaining a healthy herd can become a challenge.

Other pathogens of significance to the cattle industry include foot and mouth disease virus (FMDV) – one of the most contagious and wide-spread viruses known that can infect multiple species including humans (9); bovine leukemia virus (BLV), paratuberculosis, cryptosporidiosis, leptospirosis and brucellosis (10–12). Besides impacting cattle production, a large number of bacterial (leptospirosis, brucellosis) and some viral (BCoV, BRV) pathogens are associated with zoonoses that can cause diverse and sometimes severe diseases in humans (13).

Thus, maintaining cattle health is of utmost importance for national and global food security and human well-being. Dairy cows and calves are most significantly impacted by the industry demands whereby the young are separated from the cows almost immediately after birth and cows are bred as frequently as possible to increase milk production. Such practices lead to physiological stress and suboptimal immune function in cows and high vulnerability of their calves. Thus, an in-depth and comprehensive understanding of the immune function of these important livestock animals in the context of the current herd management practices is needed.

## Innate Immunity

In cattle, like in many other animals, the first line of defense is represented by physical barriers and mechanisms including skin and mucosal membranes, as well as elimination of invading microorganisms by coughing, sneezing, vomiting and diarrhea. Besides forming mechanical barriers of the respiratory, gastrointestinal and urogenitary tracts, epithelial cells secrete a number of antimicrobial factors, including antimicrobial peptides and defensins, and thus play an important role in the innate immune response. The other known critical cellular components of the innate immune system in cattle include neutrophils; natural killer cells, NK; dendritic cells, DC; gamma delta T cells (γδT); mucosa-associated invariant T cells (MAITs); macrophages, Mϕ, and granulocytes. While the majority of information on their development and function comes from human and mouse studies, the available data for cattle were derived from studies on mastitis, tuberculosis, BVDV, FMDV, BHV-1, and BRSV (9, 14–16). Unique to cattle, newborn calves have unusually high numbers of circulating γδT cells (up to 60% of the lymphocyte pool), and γδT cells of ruminants express WC-1 antigen whose function is unknown [may act as microbial/pathogens pattern recognition (PPR)] (14). This can be a compensatory response that balances out the immaturity of neutrophil, macrophage and DC functions in neonatal calves (17). With the exception of NK cells, the presence of innate lymphoid cells (ILCs) – another important subset of the innate immune cells - has not been confirmed in cattle thus far (9).

These diverse immune cell subsets are equipped with PPR molecules that interact with pathogen-associated molecular patterns (PAMPs) during the initial stages of the immune response (14). The best characterized among PPRs are toll-like receptors (TLRs). Ten TLRs with diverse and sometimes overlapping PAMP affinities have been confirmed in cattle (18). TLR1 recognizes triacyl lipopeptide of mycobacteria; TLR2 - peptidoglycans of gram-positive organisms and lipoarbinomannan of mycobacteria and zymosan of fungi; TLR3 - dsRNA; TLR4 – lipopolysaccharide, LPS; TLR5 - flagellin; TLR6 – diacyl-lipopeptides of mycoplasma; TLR7 and 8 - single-stranded RNA (ssRNA); TLR9 - CpG; while TLR10 function and affinity have not yet been fully assessed (18). Prior to binding to TLR4, LPS, a bacterial component of *H. somni*, *M. haemolytica*, and *P. multocida*, interacts with LPS binding protein, soluble CD14, and the TLR4 cofactor MD2. TLR4 also recognizes and binds the F protein of RSV. In addition to TLR3,7-9, BHV-1, PIV-3, BRSV, and BVDV can recognize and interact with cytosolic viral pathogen recognition receptors, retinoic acid–induced gene-I (RIG-I), and melanoma differentiation associated gene-5 (MDA-5). These interactions lead to TLR-independent activation of NF-kappa B and IRF-3 and -7 *via* mitochondrial antiviral signaling adaptor (MAVS), interferon-beta promoter stimulator (IPS-1), virus-inducing signaling adaptor, and Cardif (14).

Upon activation of the local effector cells (endothelial cells, epithelial cells, Mϕ, and DCs) of the innate immune system by inflammatory cytokines [IL1, tumor necrosis factor alpha (TNFα), and IL6], they produce a number of cytokines and chemokines that attract migration of neutrophils and monocytes into the affected area. They in turn signal to recruit to DCs and NK, T, and B cells. In contrast, IL4, IL10, and IL17 actively promote the resolution of the inflammatory cascade (19).

Bovine mast cells – a heterogenous group of cells in mucosa, skin, mammary gland and other organs that play a pivotal role in allergic and inflammatory responses by secreting biologically active substances including histamine, leukotrienes, platelet-activating factors (PAF) and prostaglandins (20–22). While immunohistochemical and functional heterogeneity of mast cells in bovines is poorly characterized, it is known that their distribution and frequency vary per anatomical site, with age and animal health. Evaluation of mast cell functions associated with chymase and tryptase production has further confirmed their anatomical compartmentalization (20).

Inflammatory chemokines include IL8 (CXCL8), GCP-2 (CXCL6), ENA-78 (CXCL5), Gro (CXCL1-3), IP-10 (CXCL10), I-Tac (CXCL11), RANTES (CCL5), MIP-alpha (CCL3) and -beta (CCL4), MCP 1-5 (CCL7, 8, 12, 13), and eotaxins 1-3 (CCL24, 26) depending on the stimulus with receptors that include CXCR1, 2, and 3 and CCR1, 2, 3, and 5. Lymphocytes that home to mucosal tissues use a different set of chemokines (e.g., naïve cells express CCR1-10, CSCR1-3 and memory cells express CCR8-10, CSCR1, 2, 4, and 5). Chemerin acts as a chemotactic factor for DCs and macrophages.

Oxylipids are another important class of immune molecules derived from cellular lipids that regulate the onset, magnitude, duration and resolution of the inflammatory response. Oxylipids are synthesized from polyunsaturated fatty acids [n-6 (omega-6) linoleic and arachidonic acids or the n-3 eicosapentaenoic and docosahexaenoic acids] (23). These fatty acid substrates are oxidized non-enzymatically by reactive oxygen species (ROS) or enzymatically by different cyclooxygenases, lipoxygenases, and cytochrome P450 producing a variety of oxylipids including prostaglandins, thromboxanes, leukotrienes, and lipoxins (24).

Natural Abs (NAbs) are an important humoral component of innate immunity. They are mostly IgM (and some IgG and IgA) Abs produced without antigenic stimulation by B1-B cells and play an essential role in primary immune response (25). A high proportion of NAbs binds to PAMPs with relatively low affinity. Complement activation by the classical pathway is one of the most important NAb functions.

Complement is another significant mechanism of innate immune defense. It consists of a group of proteins (C1-C9) present in serum in an inactive form that is activated by antigen-Ab complexes (classical pathway) or by some carbohydrates (lectin pathway) or by a variety of surfaces that are not protected by natural inhibitors (alternative pathway). The classical pathway is initiated by the activation of C1, and the alternative pathway by the C3, in an enzyme cascade order (26). Coating of bacteria or virus infected cells with fragments of the components C3 and C4 lead to their ingestion by phagocytic cells that possess receptors for these opsonins (27). Apart from its direct antimicrobial effects, complement maintains Igs in soluble form by limiting the formation of harmful immune complexes and Ig precipitation.

Thus, while sharing numerous similarities with other species, the bovine innate immune system possesses some unique features that likely together with the distinct anatomy of ruminants contributes to their resistance (rumen) or increased susceptibility to bacterial and metabolic diseases.

## Adaptive Immune Response

Bovine plasma cells produce at least five heavy chain classes (IgM, IgG, IgA, IgD and IgE), with three IgG subclasses (IgG1, IgG2 and IgG3), two IgM subclasses (IgM1 and IgM2) and two light chain types (λ and κ) (28). While the functions of IgM, IgA, IgG1 and IgG2 molecules are well-studied, those of IgG3 and IgD have not been evaluated in depth due to their later discovery. In contrast to many other animals, cattle only express a limited number of variable Ig gene segments, and it is thought that Ig diversity is achieved *via* frequent recombinations and endogenous mutations in the CDR3 region (29). Additionally, an unusually long CDR3 results in “microfolds” allowing bovine Igs to bind antigens that would otherwise be inaccessible (30). Immunoglobulin functions include neutralizing antibody (Ab, complement activation, Fc receptor-mediated phagocytosis, and Ab-dependent cellular cytotoxicity. Differences exist between different cattle breeds in relation to immunoglobulin quantities (31). IgG1 and IgG2 are highly important, with IgG1 being the most abundant in cow colostrum. IgG is important for virus and toxin neutralization and bacterial agglutination and opsonization. In cattle, IgG1 is known to be a less potent opsonin than IgG2 (32). IgM is a pentameric molecule important for bacterial agglutination, complement fixation and opsonization and is more restricted to intravascular spaces because of its size. IgA is predominant in many secretions, but present in low quantities in bovine serum (31). It is important for antiviral defense in the upper respiratory and gastrointestinal tracts.

Although methods for analysis of the clonal composition of T cells have been used extensively to gain a better understanding of the mechanisms of T cell responses in humans and mice, this knowledge is more limited for bovine T cells (33). The antigen specificity of CD4+ and CD8+ T cells is determined by the αβ T cell receptor, which binds to peptides in association with MHC class II and class I molecules, respectively (34). Most CD4+ T lymphocytes function to promote cell-mediated immunity and Ab production, while certain CD4+ Th lymphocytes are capable of lysing appropriately sensitized target cells. Most CD8+ T lymphocytes function as effector cells of direct target cell lysis and are known as classical cytotoxic T lymphocytes (CTL), but some may function as non-lytic suppressor cells (35, 36). Major α/β T cell subsets found in other species were also identified in cattle. They include: T killer (CD8+), T helper (CD4+, Th1, Th2 ad Th17) and T regulatory (CD4+/CD8+CD25+) cells, and their functions have been studied in relation to some infections and post-vaccine immune responses (37, 38). Earlier data suggested that cytokine-mediated regulation of Th1/Th2 cell responses in bovids may be more complex and distinct from that observed in mice (39). Specifically, parasite-specific bovine Th cells produce IL4 and IFNγ, while IL2 and IL10 expression is not restricted to IFNγ or IL4-producing cells, respectively. Also, IL4/IL10 and IL12 do not selectively exert their suppressive or stimulatory effects on Th1-like cells. Nevertheless, some studies demonstrated that development and maintenance of a Th1 IFNγ response can be associated with a greater control of some infections (*M. bovis*) (40).

Besides their critical role in shaping B cell responses, several studies emphasize the importance of bovine T cell responses that contribute to clearance of infections (for example *Cryptosporidium parvum*) in the absence of humoral/Ig responses (41). Of interest, memory CD4+, CD8+, and γδ T cells are detected in calves in the presence of maternal Abs even in the absence of an active Ab response (38).

Thus, the bovine adaptive immune responses are driven by a combination of common and unique aspects that need to be considered when developing age- and herd-specific preventative and therapeutic strategies.

## Mucosal Immune System

The mucosa includes 4 distinct major components: (1) microbiome; (2) mucous layer, (3) mucosal epithelial barrier, and (4) immune cells of the mucosa-associated lymphoid tissues [MALT; lamina propria (LP), Peyer’s patches (PP) in the gut and diffuse mucosal lymphocytes] (42, 43). The microbiome is critical for immune development of young calves as well as immune defense and health maintenance of cattle of all ages (43). While the role of the rumen in mucosal immunity has not been yet clarified, it is established that bacterial richness and diversity were the highest compared with all other gut sections (42, 44). Bile acids (BA) in the small intestine represent another important mechanism of intestinal defense (45) and the gut microbiota plays a role in their metabolism. Bile acids inactivate enveloped viruses and some enteropathogenic bacteria; however, enterococci and *Bacteroides* spp. can degrade them, while commensal bacteria metabolize BA in the colon (45). The mucous barrier consists of the mucous and mucins (secreted by Goblet cells), antimicrobial peptides (AMPs; defensins, REGIII, lactoferricin), as well as IgG and secretory IgA (transported from LP). Epithelial cells (ECs; enterocytes in the gut and ciliated ECs in the lung) line the gastrointestinal (GIT), reproductive (RepT), urinary (UT) and respiratory (RespT) tracts and express tight junction proteins forming the mucosal epithelium (ME). If the tight junctions break down, the epithelium becomes leaky leading to systemic inflammation. This condition is best known as “leaky gut” but can be observed in RepT and RespT. Besides providing mechanical segregation, the ME functions include secretion and absorption (in the gut), fetal development (in the uterus), oxygen exchange and clearance of foreign substances and pathogens (in the RespT), and innate immune response. Bovine ECs express the whole repertoire of TLRs (1–10, 46). Based on luminal stimuli, ECs can produce pro-inflammatory (IL1α, IL8, and TNFα) or regulatory cytokines (IL10 and TGFβ) (42, 47). The microbial components stimulate ME to produce serum amyloid A, which stimulates DCs to activate other important mucosal Treg cell, Th17 cells that produce high amounts of IL17A and ILnd moderate amounts of IL22 and IFNγ (48, 49). The latter are critical for mucosal protection and repair as well as for defensin production (e.g., REGIIIγ and REGIIIβ) (42).

While the information regarding rumen-associated lymphoid tissues is limited, rumen fluid leukocyte populations (including monocytes, T and B cells) are likely to play a significant role in regulation of cattle immune and biochemical parameters (50). So, CD45 gene expression in rumen fluids (indicative of leukocyte infiltration) negatively correlated with ruminal pH, while the frequencies of B cells (as well as numbers of total IgG and IgM) were negatively affected by ruminal pH and high concentrations of volatile fatty acids. Additionally, rumen health disorders (e.g. subacute ruminal acidosis), were shown to cause ruminal dysbiosis breaching the epithelial barrier and leading to inflammation (51).

Organized MALT – the induction site of mucosal immunity - is widely distributed in the mucosa throughout the body allowing for antigen sampling from mucosal surfaces. It is composed of gut-associated lymphoid tissues (GALT), bronchus-associated lymphoid tissues (BALT), nasal (nasopharynx)-associated lymphoid tissues (NALT) and lymphoid tissues in the RepT, UT, mammary gland(s), lacrimal glands and salivary glands. These lymphocyte aggregates or follicles (also known as lymphoid follicles [LF] or PP in the gut) of B cells, T cells, and DCs and macrophages antigen presenting cells (APCs) are covered by specialized epithelial cells called dome or M cells [found in BALT, GALT and in uterus] (52–54). The GALT is the largest lymphoid organ and the largest body surface in contact with a great diversity of food and microbial antigens. The M cells (present in MALT) pinocytose antigens and transport them across the ME, where they are processed by APCs and presented to T and B lymphocytes (54, 55). Then, antigen-stimulated mature T cells and B cells act together to produce IgA. Some of them express special molecules in their membranes (homing receptors) and leave the submucosal lymphoid tissue and enter the bloodstream; they can further exit the bloodstream through high endothelial venules and translocate to LP. Ultimately, this local stimulation results in memory T and B cells that can migrate to the nearby and distant mucosal and other tissues, which is known as “common mucosal immune system” (42). A part of this “common mucosal immune system” - the bovine entero-mammary link (or gut-mammary axis) is critical for neonatal calf survival and health (45). This endogenous entero-mammary pathway not only allows lymphocytes to traffic to the mammary gland, but also transports some bacterial components during lactation in the cow (56).

A better understanding of the mucosal immune response and systematic evaluation of bovine vaccine efficacy based on immunization route will help to identify the pathogens that may be cleared more efficiently with mucosal *vs*. parenteral vaccines.

## Systemic Immunity

While the mucosal immune responses are more important for the clearance of most pathogens (especially enteric and respiratory), for a number of viral and bacterial agents associated with systemic, chronic or lymphoid organ infections, protective immunity and pathogen clearance are unachievable without a robust systemic immune response. These pathogens include (but are not limited to): *Staphylococcus aureus*, FMDV, BRSV, *Haemophilus somnus* and *Mycoplasma bovis* (57–60). Studies of immune responses to these pathogens identified some commonalities as well as distinct systemic responses. IgG1 and IgG2 Ab responses predominant in serum (in contrast to IgA in various mucosal secretions), as well as potent and balanced CD4+ and CD8+ T cell and IFN type I responses in systemic immune organs and blood, were associated with improved protection. Another pathogen common in cattle, *Leptospira interrogans (L. interrogans)* enters the hosts through chafed skin or mucosa and then is disseminated in blood, potentially leading to renal infection (61). At the disease onset, symptoms are non-specific leading to it being underdiagnosed and poorly controlled by vaccines. Intraperitoneal priming with a TLR2/NOD2 agonist was shown to induce a sustained protective systemic innate immune response characterized by enhanced pro-inflammatory cytokine, chemokine and nitric oxide production by macrophages independent of the presence of B and T cells (62).

While most recent data suggest the importance of the systemic immune responses in some infections, it is also evident that tissue-specific immune responses may play important roles specific for each pathogen which requires further evaluation.

## Pathogens Associated With Immunopathology

Several important bovine pathogens have evolved mechanisms allowing them to subvert the immune system and establish persistent infections. These mechanisms are diverse and not completely understood which makes the use of classical vaccination and other approaches to control these pathogens challenging and sometimes impossible. Below we will summarize the strategies used by some of these pathogens to suppress host immune responses, as well as the mechanisms they use to modify themselves or their location in the host to block the recognition by the host immune system.

BVDV has evolved unique mechanisms to establish persistent infection characterized by immune tolerance of the non-cytopathogenic (ncp) variant (63). Due to its immunosuppressive effects on both the innate and adaptive immune systems, BVDV acts as a major predisposing factor for BRD. Interestingly, ncpBVDV suppresses production of IFN type I and pro-inflammatory cytokines in bovine alveolar macrophages whereas its cytopathogenic counterpart triggers this response. This suppression leads to the decreased phagocytic activity. Additionally, cells infected with ncpBVDV are also resistant to induction of interferon by dsRNA, a potent inducer of IFN type I. Further, *in vitro* infection of monocyte-derived macrophages with both cytopathic and ncpBVDV suppresses responsiveness to TLR2, TLR3, TLR4 but not TLR7 ligands (64). Because IFNα production is critical for initiation of the adaptive immune response, its inactivation by ncpBVBDV may represent the key mechanism affecting both innate and adaptive immunity. Similar to *in vitro* findings, *in vivo* BVDV infection also modulates the capacity of monocytes and macrophages to respond *via* TLR4 (65).

Another example of immunopathological response associated with bovine viral pathogens is bovine leukemia virus (BLV). It is characterized by increased numbers of CD4+CD25+Foxp3+ Treg cells that produce higher levels of TGFβ resulting in reduced production of IFNγ and TNFα by CD4+ T cells and impaired NK cell function (12, 66). These immunological impairments lead to increased susceptibility to opportunistic infections. Additionally, the production of antiviral cytokines (including IFNγ, IL2 and IL12) by lymphocytes and their proliferative ability in response to BLV was also significantly reduced in cattle with persistent lymphocytosis (67). In contrast, it was demonstrated that the protective Th1 response against *Mycobacterium avium* subsides later in the course of the infection, while the non-protective Th2 response becomes prominent. Recent data demonstrated that T cell unresponsiveness, rather than Treg activity, is driving this Th1-to-Th2 immune shift (68).

Recent studies have improved our understanding of exhaustion and dysfunction of antigen-specific T cells in chronic infectious diseases of cattle. They demonstrated that up-regulation of surface expression of immunoinhibitory receptors, such as programmed death 1 (PD-1), lymphocyte activation gene 3 (LAG-3), T-cell immunoglobulin and mucin domain-containing protein 3 (Tim-3) and cytotoxic T-lymphocyte antigen 4 (CTLA-4) by bovine T cells play a critical role in immune exhaustion and disease progression in the case of BLV infection, Johne’s disease (caused by *Mycobacterium avium*) and bovine anaplasmosis (11, 69–72).

Of interest, while some FMDV infected ruminants can clear the virus within 1-2 weeks, some remain persistently infected for up to 3 years (73). This dichotomy and how FMDV evades cytotoxic T cell responses are not completely understood. Thus, further studies to understand this and to determine how the carrier status and FMDV-associated immune modulation can affect cattle susceptibility to other pathogens are warranted.

These pathogens can further compromise cattle health predisposing them to secondary infections due to immune suppression. Strict biosecurity measures (whenever possible) combined with vaccines and nutritional interventions need to be implemented to reduce prevalence of these pathogens.

## Immune System and Microbiome

As in most other mammals, bovine fecal microbiota is dominated by the 5 following phyla: *Firmicutes* (the most prevalent, 63.84%-81.90%), *Bacteroidetes* (8.36%-23.93%), *Proteobacteria* (3.72%-9.75%), *Fusobacteria* (0.76%-5.67%), and *Actinobacteria* (1.02%-2.35%) (74). Similar to findings from other species, fecal microbial diversity in cattle is associated with diet, age, disease status and growth rates, and increased abundance of *Faecalibacterium* spp. is suggested to promote health and growth (74).

The development and establishment of the gut microbiome is a dynamic process that can be influenced by several internal and external factors. Internal (=host-related) factors include functional maturity of the gut and immune system, biliary secretions and repertoire of bacterial mucosal receptors (75, 76). The list of external factors is broader including everything in the calf environment including the calf nutritional status, vaginal, fecal and milk microbial composition of the cow, antibiotic use, etc. (43). As the calf grows, the gut bacterial composition develops quickly culminating in a “climax” community maintaining the anaerobic environment (77).

The rumen – an anaerobic and methanogenic forestomach - houses an abundant and complex microbiota (~10^10^–10^11^ cells/ml and over 200 species) responsible for the remarkable ability of cattle to transform indigestible plant mass into essential nutrients (78). Bacteria are the most abundant microbes in the rumen and their composition is determined by a number of factors including the diet, energy requirements, and resistance to certain metabolic byproducts toxic to some species. Several studies demonstrated that rumen bacteria of animals fed high forage or high grain diets mainly consisted of Gram negative or Gram positive bacteria (including *Lactobacillus)*, respectively, while increased proportion of corn silage resulted in increased *Prevotella* and decreased protozoal abundance (79, 80). Numerous studies demonstrated that a large microbial component remains uncultured, while fundamental diet-driven differences were noted in the glycoside hydrolase content (81). Another recent study emphasized the importance of the ruminal microbiota and suggested that different breeds of dairy cows have different metabolic, immunological and performance traits (82).

Central (e.g., bone marrow and thymus) and peripheral (e.g., lymph nodes, spleen, and MALT) organs of the calf immune system develop in prenatal and postnatal periods with maternal and calf microbiota playing a significant role during both periods (43, 83). In the first 24-36 hours of life, calf gut permeability decreases significantly due to the increase in expression of the tight junction (TJ) proteins (occludin, claudins, zonula occludens, and junctional adhesion molecules). Although, the exact mechanisms are not known, interactions between the ME and some bacteria (e.g., *Lactobacillus spp* and *Bifidobacterium* spp) or bacterial metabolites upregulates TJ expression promoting intestinal barrier integrity (84). The production of mucus – another immune defense barrier - is stimulated by the presence of commensal bacteria (85). As summarized by Gomez and colleagues, other evidence accumulated in various animal models indicates that commensal microbiota stimulates enterocyte turnover and metabolic activity, enhances production of AMPs by enterocytes and Paneth cells and secretory IgA production (43). Low levels of secretory IgAs are associated with bacterial expansion leading to systemic inflammation and/or diarrhea (86). While over-proliferation of *Enterobacteriaceae spp* has been associated with diarrhea in calves (87), the role of IgA secretion in this condition is unknown and warrants further investigation. Further, expression of TLR2 and TLR6 is downregulated with increasing age in calves which is associated with increased abundance of digesta and tissue-associated total and lactic acid bacteria (88). Expression of TLRs in the gut of calves is also influenced by gut region, which in turn can be due to regional variations in the density of microbial communities (88). It is also possible that the colonization of the calf gut by *Lactobacillus spp* and *Bifidobacterium spp* promotes a regulatory immune response (increased IL10 secretion) thereby avoiding exacerbated inflammatory responses to commensal microbiota (89). While there are now comparative studies evaluating systemic immune responses in germ-free and conventional calves, there is growing associative evidence that the microbiome-associated alterations in the immune function are observed in the gut and systemically. For example, the stress of comingling, transportation, weaning and other abrupt dietary changes also alter microbial composition in the gut which may lead to intestinal dysbiosis. Dysbiosis and the associated immune alteration can in turn predispose calves to various infectious diseases, including Johne’s disease (paratuberculosis) (90). Another example of systemic immune modulation by gut bacteria is from a study where lysozyme, lactic acid and glycopeptide (isolated from *Lactobacillus* spp) treatment decreased numbers of T helper, but not CD8+ cells, numbers of CD25+, CD38+, as well as CD69+ and CD95+ cells and increased frequencies of IL2 receptor expressing cells in cow blood. Those changes were associated with a significant elevation of somatic cell counts and decreased numbers of pathogenic bacteria in the milk of the treated cows (91).

These data suggest that intestinal dysbiosis and the associated infectious or metabolic diseases in dairy cattle may represent a significant challenge that needs to be addressed *via* nutritional adjustments or probiotic treatments.

## Immune System and Nutrition

Dairy cow nutritional status and metabolism of specific nutrients are critical for adequate immune and other cell functions. The effects of nutrition may be direct through nutrients or indirect *via* metabolites. A majority of health problems in dairy cattle occur around parturition due to the hormonal shifts and the need of adaption to the increased nutrient demands for lactation resulting in negative energy balance. Diet-associated uncontrolled inflammation is considered to be a major contributing factor to several metabolic diseases common in dairy cattle including mastitis, retained placenta, metritis, displaced abomasum, and ketosis (92). Previous studies demonstrated that over- or under-nourished cows were more susceptible to various infectious diseases compared to those with adequate nutritional status in periparturient period (93). Somewhat conflicting evidence of the associations between transient dietary restrictions, neutrophil function reduction and various inflammatory diseases in postpartum dairy cows is discussed by Sordillo (92). Provision of appropriate antioxidants including omega-3 polyunsaturated fatty acids, conjugated linoleic acid and vitamin D promotes anti-inflammatory responses (94). Deficiencies in certain micronutrients (vitamins and trace elements) are associated with an increased incidence of mastitis, retained placenta, and metritis (95). The immunomodulatory and antioxidant effects of various macro- and micronutrients that influence the incidence of health disorders in dairy cattle have been discussed in detail in several previous reviews and are briefly summarized in **Table 1** (109–113). These immunological and metabolic disturbances in lactating cows as well as dairy calf feeding practices may result is suboptimal nutrition and immune protection of neonatal calves.

**Table 1:** Role of macro- and micronutrients in the immune function of cattle.

Nutrient	Role in immunity	References
Fat/energy	Regulates cell mediated immunity and Ab response. Fat-derived fatty acid composition of immune cells affects phagocytosis, T cell signaling and antigen presentation capability	(96, 97)
Protein	Protein/amino acids are required for proliferation and maturity of immune cells. Specific amino acids (e.g. tryptophan, arginine, glutamine) are required for systemic and gut immune function	(98)
Glucose	Up-regulation of cell proliferation, differentiation, survival, chemotaxis, phagocytosis	(99)
Glutamine	Up-regulation of cytokine and reactive oxygen metabolite (ROM) production, cell division, phagocytosis, CD4 T cell proliferation	(100)
Tryptophan	Activation and maintenance of the immune response	(101)
Fatty acids	Down-regulation of IgM secretion, cytokine production, cell viability, phagocytosis, diapedesis, antigen presentation. Up-regulation of oxidative burst, necrosis, phagocytosis, cytokine and ROM production, TLR signaling	(96)
Selenium	Maintenance of the antioxidant system, enhancement of neutrophil function and neutrophil and macrophage migration	(102, 103)
Zinc	Overall immune function, antioxidant activity [integral part of superoxide dismutase (SOD)], epithelial barrier integrity, nucleic acid and protein synthesis, cell division	(98, 103)
Copper	Overall immune function, antioxidant activity [integral part of superoxide dismutase (SOD)], enhancement of interferon production	(98, 104)
Iron	Antioxidant defense (essential component of catalase), energy and protein metabolism, oxidation–reduction reactions	(105)
Manganese	Overall immune function, antioxidant protection (integral part of SOD), carbohydrate and lipid metabolism	(106)
Chromium	Regulation of cell-mediated and humoral immune responses, upregulation of blastogenic response, enhancement of cytokine (IL2, IFN, and TNFα) production by mononuclear cells, Ab production	(98)
Vitamin A, β-carotene	Overall immune function, upregulation of lymphocyte proliferation	(103)
Vitamin B	Antioxidant defense, upregulation of lymphocyte proliferation	(98)
Vitamin D	Antioxidant defense, down-regulation of inflammation	(107)
Vitamin C	Antioxidant defense, down-regulation of inflammation	(108)
Vitamin E	Lipid soluble antioxidant, enhancement of neutrophil function, increase of production of IL1 and major histocompatibility (MHC) class II antigen expression	(102, 103)

## Immune Function in Pregnancy and Early Parturition

**Figure 1:**
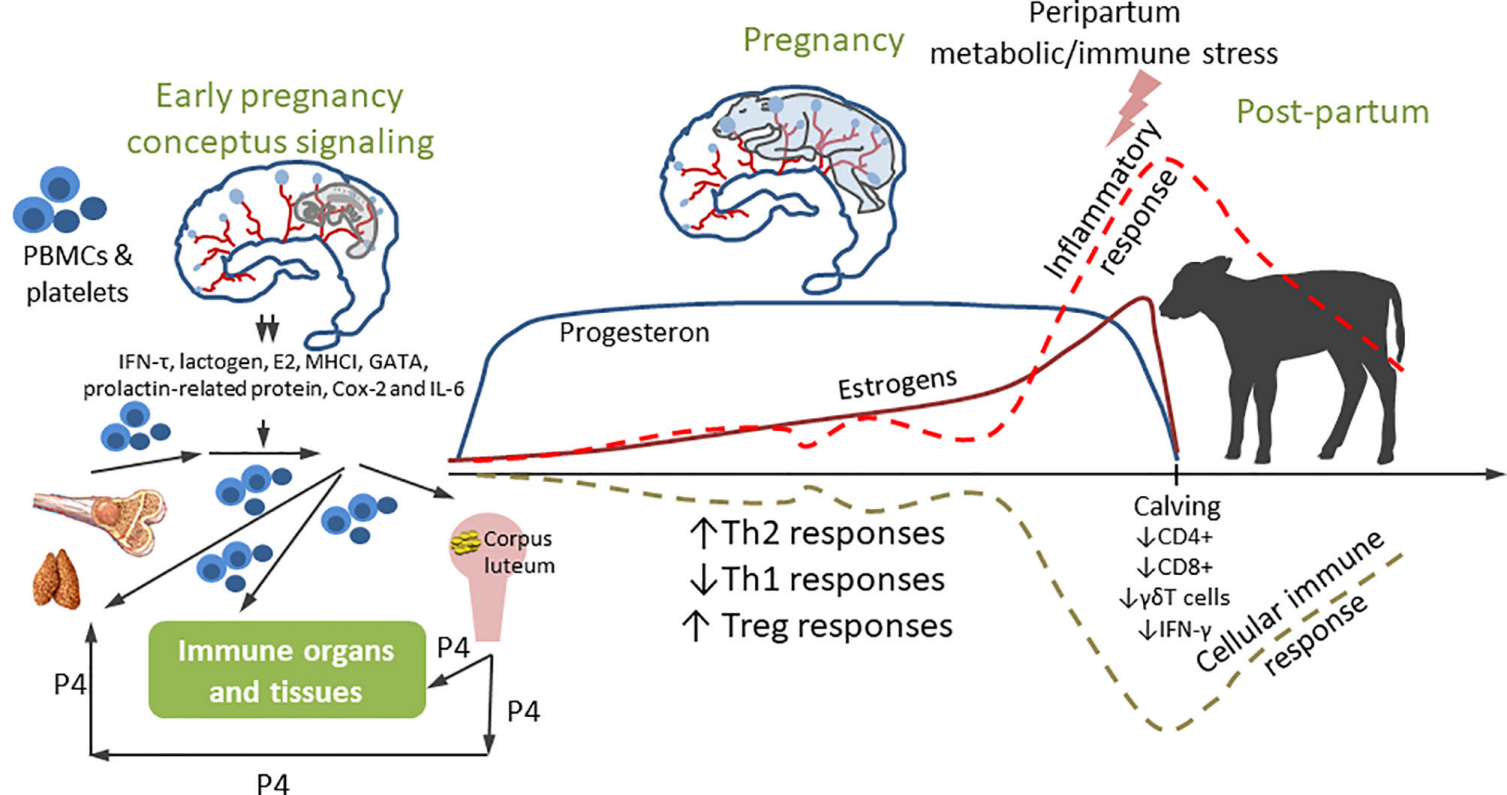
Immune modulation during pregnancy. Early conceptus signaling modulates local and systemic immunity. The peripheral blood mononuclear cells (PBMC), platelets and cell-free DNA from bone marrow/thymus modulated by high level of P4 from corpus luteum enter blood circulation. Then, the PBMC, platelets and cell-free DNA while migrating through blood circulation to endometria, are controlled by IFNT and other conceptus immune factors. Then the functionally changed PBMC, platelets and cell-free DNA re-enter blood/lymph circulation and traffic to effector cells affecting the function of the immune organs and non-immune organs including ovary. This early signaling shifts immune environment to Th2 to maintain the pregnancy. Inflammatory responses sharply increase, and cellular immune function is down-regulated around parturition.

The immune mechanisms discussed above play critical roles at the maternal-fetal interface and in the neonatal periods. Importantly, pregnancy hormones induce changes in immune cell populations and functions to promote immune tolerance, reduce expression of major histocompatability proteins by the trophoblast, tissue remodeling, and angiogenesis (114). In early pregnancy in cattle, complex hormonal and immune shifts occur: the conceptus blocks the luteal regression to maintain progesterone production that prevents the fetus from destruction by the maternal immune system. Recent studies evaluated immune regulation of the maternal uterus, peripheral blood mononuclear leukocytes and corpus luteum induced by IFNτ, the primary pregnancy recognition signal in cattle (115). Frequencies of endometrial NK cells, CD8+ T cells, macrophages and DCs as well as IL15 and IL10 cytokine levels are also markedly increased in early pregnancy (114). The findings suggested that the bovine embryo triggers an anti-inflammatory response in immune and epithelial cells. Furthermore, expression of indoleamine 2,3 dioxygenase (IDO, converts tryptophan to kynurenine altering immune function) is increased in the first trimester in dairy heifers (114). IDO activates the aryl hydrocarbon receptor (abundant in bovine uterus), thus, inducing downstream tolerogenic mediators. Pregnancy is also associated with increased expression of proteins inhibiting immune activation and inducing lymphocyte tolerance, including programed cell death ligand-1, lymphocyte activation gene-3, and cytotoxic T-lymphocyte associated protein-4. There is also evidence of enhanced TLR expression and macrophage recruitment and activation in the cow endometrium early in pregnancy; however, the reasons for the latter are not fully understood (116). Besides IFNτ, placental lactogen, pregnancy-associated proteins, prostaglandin E2, non-classical MHC class I, GATA transcription factors, prolactin-related protein, Cox-2 and IL6 are secreted by the conceptus in early pregnancy. It was suggested that IFNτ (and other conceptus signaling factors, **Figure 1**) may be involved in maternal systemic immune regulation through modifying peripheral blood mononuclear leukocytes, platelets and cell-free embryonal DNA by the lymph circulation and blood circulation in the bovine (**Figure 1**). Besides its anti-luteolytic function, IFNτ also functions to promote uterine receptivity and embryo development. It enhances the expression of IFN-stimulated genes (including TNFα and MCP1) in the endometrial tissue of cattle (117). Maternal immune responses during embryo elongation in cattle include increased numbers of monocytes and DCs in the endometrial stroma, while MCP1/2 serve as a potent chemotactic factor for monocytes and DCs (117). Further, a subpopulation (M2) of activated macrophages and several cytokines [including IFNγ, IL4, and Leukemia inhibitory factor (LIF)] act to decrease the activation of anti-conceptus immune responses (118–120). These early modifications and consistently high levels of progesterone (P4, **Figure 1**) result in an immune balance shift toward Th2 and its maintenance until periparturient period (121, 122). At parturition, however, the ratio of Th1/Th2 should increase a rapid transition from tolerance for the fetus (high Th2) to protection against infectious agents (high Th1) (123). There is also limited evidence of partial immune suppression in periparturient cows showing that frequencies of peripheral blood CD4+, CD8+ and γδT cells and IFNγ decline, while the numbers of CD25+ T cells increase (116). B cell clonal expansion and antibody production also decrease reaching nadir around parturition (124). The week prior to and immediately after parturition is also associated with neutrophilia, eosinopenia, lymphocytopenia and monocytosis. Additionally, cows show a decrease in phagocytosis and oxidative burst activity (125). These findings suggest that there is another immunological shift occurring before calving. Heyland et al. (126) hypothesized that around parturition multiple external insults can induce a systemic inflammatory response capable of attenuating the cellular immune response (126) (**Figure 1**). Further, some findings suggest that release of fetal membranes can be an immune/hormone-mediated process because increased incidence of retained placenta (RP) is observed in cows sharing MHCI antigen specificity with their calves (127). It is well established that daily food intake decreases (~30%) as calving approaches (128), which coincides with increases in levels of pro-inflammatory cytokines (50). Dairy cows do not consume enough nutrients to meet the increased demand to support lactation which leads to a negative energy balance (NEB). NEB in turn leads to suppressed immune function or poorly controlled inflammation which may promote uterine diseases and other metabolic diseases including milk fever, ketosis, and displacement of the abomasum (113).

Thus, the immune function and its regulation during pregnancy, from the conception and through parturition are very complex and dynamic with selective suppression and up-regulation of distinct immune responses. These aspects have to be carefully considered in vaccination approaches to maintain optimal health of periparturient cows and neonatal calves. Other disease controlling strategies should also be tailored to the stage of production of dairy cows.

## The Bovine Mammary Gland Immune Defenses

The ability of lactating cows to resist invading pathogens (bacterial) and clear them is primarily dependent on the function of the mammary gland (MG) immune system, although systemic immunity also plays a role. Also, calves are born agammaglobulinemic (because there is no transplacental transport of Igs in cattle) and rely fully on consumption of colostrum and milk to ensure adequate passive immunity (83).

**Figure 2:**
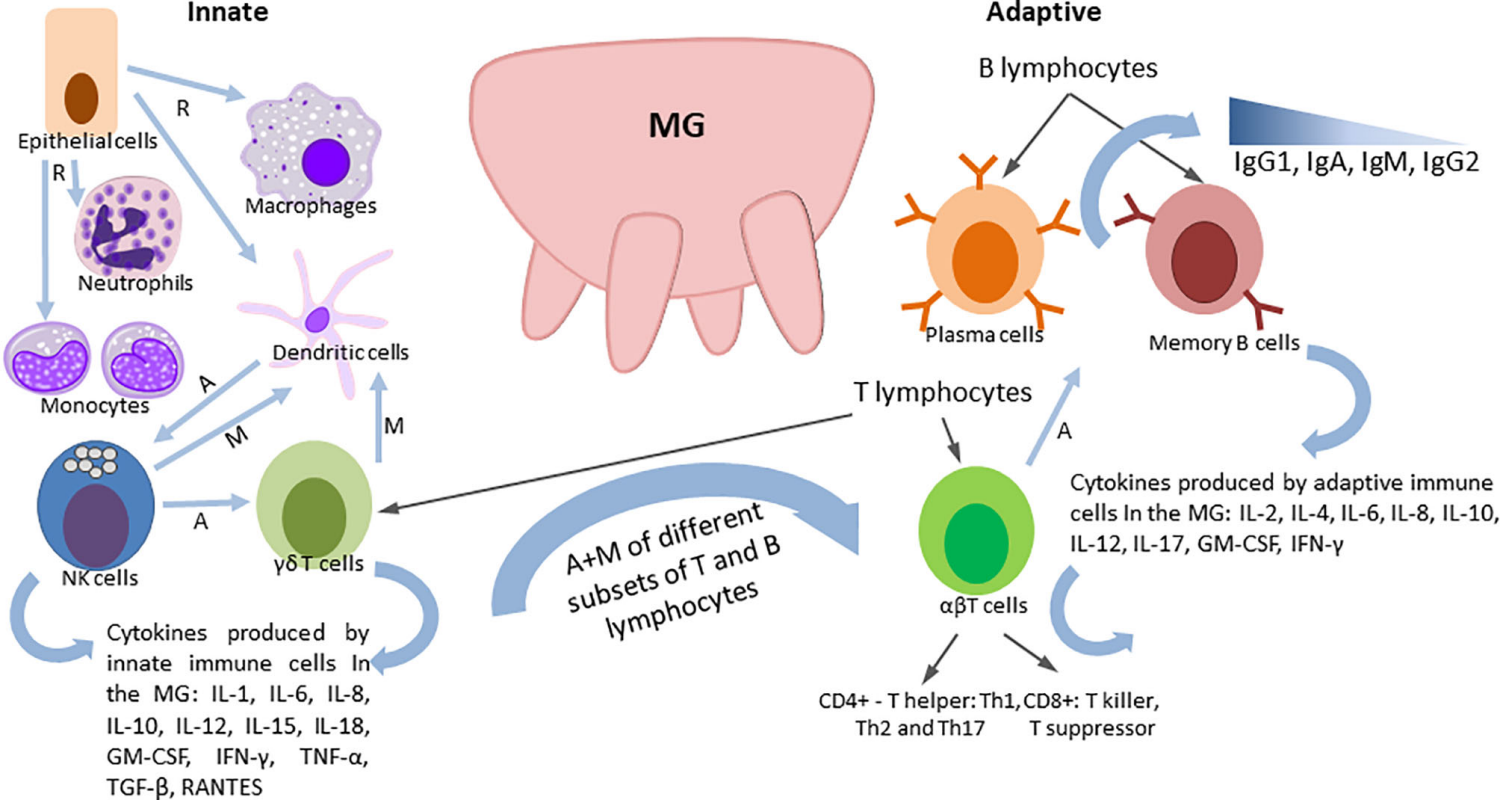
Immune cells of the mammary gland (MG), their functions and cytokines produced.

The bovine MG is equipped with a non-immune anatomical barrier, and a plethora of immune mechanisms, including coordinated action of innate and adaptive immune responses (129). Detailed reviews by Rainard (130), Ezzat Alnakip (131) and Sordillo (132) summarized the immunobiology of the bovine MG and the mechanisms of its immune defense that include common cellular and soluble immune components discussed earlier as well as some unique biochemical, mechanical and immune factors (**Figure 2**). Among the latter is the teat canal barrier with the following defense factors: contraction of the teat sphincter muscles to block bacterial penetration; bacteriostatic activity of keratin and Furstenberg rosette densely populated with leukocytes. Lactoferrin, a unique soluble secretion of the MG, is one of the best-characterized antimicrobial proteins and is the most common iron-binding protein that greatly reduces soluble ferric iron available to multiplying bacteria. Transferrin, lysozyme, lactoperoxidase and xanthine oxidase are also found in the milk of ruminants that play diverse roles in antibacterial defense (130). Major cell subsets with diverse immune functions, including epithelial cells, innate immune cells and T and B lymphocytes (**Figure 2**) are present in the MG. These cells interact directly or *via* soluble components to ensure optimal protection of the udder against invading bacteria and to provide passive protection to the calves with colostrum/milk.

Of all TLRs, TLR2 and TLR4 are of particular importance to MG defense because these receptors recognize PAMPs associated with gram-positive (peptidoglycans) and gram-negative (LPS) mastitis-causing pathogens, including *Staphylococcus aureus*, *Streptococcus uberis*, and *Escherichia coli* (133). While rapid and robust inflammatory immune responses can efficiently block or clear infection, their increased duration can lead to excessive tissue damage. This emphasizes the importance of the MG immune regulatory function that leads to the cessation of proinflammatory mediator synthesis and their catabolism. Cytokines IL4, IL10, and IL17 and oxylipids (regulating microvasculature and pro-/anti-inflammatory responses) play a critical role in the resolution of inflammation (19). The contribution of the complement system to the MG immune defense has been discussed in detail (27, 134). The classical pathway is not functional due to the lack of C1q, but the alternative pathway can operate, resulting in: 1) deposition of opsonic C3b and C3bi on bacteria, and generation of the pro-inflammatory fragment C5a (130). Natural Abs that cause bacterial opsonization are another component of innate humoral defenses. While generally, opsonic Abs belong to the IgG2 and IgM isotypes, the majority of the opsonic Ab in cow serum and milk of cows are IgM (130). Virus or bacteria specific immunoglobulins are the most important soluble humoral factors of the adaptive immune defense of the MG, linking the cellular and humoral immune system. Four classes of Igs (IgG1, IgG2, IgA, and IgM) influence MG antibacterial immune defense and play distinct roles depending on the lactation stage. IgG1 is the most abundant Ig class in bovine milk and colostrum (131, 135), while IgG2 levels increase substantially in inflammation (26) (**Figure 2**). Immunoglobulins in milk may be blood derived or produced *in situ* by antigen activated plasma cells, which traffic to the udder from the blood (136). Thus, unlike in monogastric animals, the dominant Ab (IgG1) in bovine colostrum/milk is actively transported from the peripheral blood (and not the gut), which allows for the use of parenteral vaccines in pregnant cows to boost serum IgG1 Abs with their subsequent transfer into milk and colostrum (137). The active IgG transport is mediated by neonatal Fc receptor, which is an intracellular, bidirectional and pH-dependent process (138).

A depression of several immune functions that has been reported around parturition (in part regulated by increased level of blood glucocorticosteroids) manifests by a higher prevalence of clinical mastitis and other diseases during this period (139). An increase in IL2 secretion from late gestation to parturition emphasizes the immunological function (antiviral protection) of the bovine MG (140).

These unique aspects of bovine immune responses allow to provide protection to neonatal calves *via* parenteral maternal vaccination. Vaccination strategies should be further optimized to select optimal vaccination timing, vaccine types, doses and adjuvants.

## Passive Immune Protection of Neonatal Calves

Dairy calves provide replacement animals critical for the future of the dairy operation. Passive immunity in calves is assessed by quantifying the levels of serum IgG or total protein during the first 7 days of age. Beef and dairy calves feeding is managed differently, beef calves are allowed to suckle, while dairy calves are separated from dams at birth or soon after, fed colostrum and placed on milk replacers. These differences in colostrum management may affect early immune development of calves. Whole blood immune transcriptome analyses demonstrated that there was a surge in pro-inflammatory cytokine expression in dairy calves, while sucking beef calves had increased expression of genes associated humoral immunity maturation by 7 days of age (141). Cow colostrum and milk provide key nutrients and passive protection to neonatal calves. Colostrum/milk components also include casein, lactoferrin, whey proteins and lactoperoxidase as well epithelial and immune cells (macrophages, T and B lymphocytes). These cells cross the neonatal intestinal barrier and populate peripheral and central lymphoid tissues promoting the calf immune development (142). Bovine colostrum also contains potent bioactive components that promote growth (EGF and TGFβ) and counteract pathogens (proinflammatory cytokines: IL1b, IL6, TNFa and IFNα), enhance lymphocyte function and promote maturation of the neonatal intestinal immune system (143). Cytokine concentrations in bovine colostrum are much higher than in mature milk, which contribute to the production of secretory IgA as well as Th1 and Th2 responses (144). Colostrum also contains maternal immunoglobulins and immunomodulatory factors that suppress development of active immunity (145). Thus, in addition to nutrition, mammary gland secretions play at least two more important roles: generation of tolerance to food antigens and commensal microbiota while promoting immune development and immune response to pathogens.

Because passive transfer of lactogenic soluble and cellular components from the mother to the calf prior to the gut closure (cessation of transport of macromolecules across the gut barrier) is the main means of anti-infectious protection immediately after birth, its failure (FPT) due to compromised maternal health, late calf feeding or feeding poor quality colostrum is associated with >30% of mortality in pre-weaned calves (146). While low colostrum intake at birth is a risk factor for FPT for all calves, those from younger (especially primiparous) cows have lower serum Ig concentrations compared to those born to older cows, which also contributes to increased morbidity and mortality. Supplementation of commercial colostrum represents a viable strategy to alleviate the negative effects of the passive transfer failure (147). A majority of dairy calves are reared on pasteurized waste milk and milk replacers (148), and they are at higher risk suffering negative health sequalae due to FPT. Recent studies demonstrated that 2 *vs* 1 colostrum feedings after birth have resulted in decreased risk of FPT, reduced morbidity and improved growth rate (149, 150). Finally, the protection from the passive immunity transferred to calves peaks 1–2 days and then starts declining. Currently available commercial milk replacers are generally made of skim milk powder, vegetable or animal fat, buttermilk powder, whey protein, soy lecithin and vitamin-mineral premix (151). While their nutritional value has improved over the past decades, they remain deficient in immunological and growth/development promoting components leading to higher rates of infectious diseases.

These observations emphasize that the current feeding practices of pre-weaned dairy calves should be further optimized, including introduction of multiple colostrum feedings. Similarly, vaccination strategies of pregnant cows require in-depth evaluation to ensure optimal maternal health and passive protection of their offspring.

## Existing Vaccines and Their Efficacy

Vaccination is a critical component of beef and dairy cattle health management. Effective vaccination programs require in-depth knowledge of the circulating pathogens, their pathogenesis and immune responses in cattle, selection of the optimal timing and consideration of host factors such as age, health status, reproductive status, and external stressors.

Use of modified live-virus vaccines against respiratory pathogens in cattle upon feedlot arrival has been consistently recommended; however, emerging data suggest that it might not be optimal (152). This is because the stress associated with transportation leads to increased levels of cortisol and other pro-inflammatory factors that compromise vaccine efficacy. Thus, it is likely that giving the required vaccines prior to cattle shipment would be more beneficial; however, studies are needed to confirm this. Further, while benefits of maternal vaccination for improved calf health and survival are established (153–155); the optimal gestational age is not determined in cattle. Recent studies in pregnant swine demonstrated that second (but not first or third) trimester vaccination against porcine epidemic diarrhea virus resulted in optimal immune responses and health/survival outcomes in sows and their piglets (156).

Significant efforts were made in the last two decades to evaluate the efficacy of cattle vaccination against tuberculosis using human Bacille Calmette-Guérin (BCG, Mycobacterium bovis) vaccine, and the results have been promising (157–159). A meta-analysis of the effectiveness of vaccination of cattle with commercially available viral vaccines for mitigation of morbidity and mortality from BRDC has demonstrated variable results (160). While BHV-1 and BVDV vaccines decreased the risk of BRDC mortality/morbidity; experimental trials showed no differences between BRSV and PI3 vaccinated and control calves in reducing BRDC morbidity or mortality risk (160).

While many licensed vaccines and preventative therapeutic products for cattle are available (**Table 2**), there is still surprisingly limited, but emerging data on the efficiency of the existing vaccines for cattle in different production environments. Thus, the majority of vaccines, while showing some efficacy require further optimization and a better understanding of the bovine immune response and health status in different ages and production groups. Further, the optimal timing for vaccination of pregnant cows is not determined and coverage for some vaccines remains insufficient. Additionally, oral vaccination with attenuated pathogens may provide stronger and more lasting immunity. Finally, detailed examination of molecular mechanisms utilized by each pathogen (including immune suppressive mechanisms discussed below) and environmental factors is essential.

**Table 2:** USDA licensed antiviral and antibacterial vaccines and preventative therapeutic products for cattle.

Vaccine/type	Licensed producer	Effectiveness
Autogenous vaccine, killed virus, autogenous bacterin	SolidTech Animal Health	Variable (161)
Autogenous vaccine-autogenous bacterin	Biomune Company, Cambridge Technologies, Colorado Serum Company, Elanco US, Hennessy Research Associates, Huvepharma, Kennebec River Biosciences, Newport Laboratories, Phibro Animal Health, Texas Vet Lab	
BHV-1 live vaccine; H. somnus, M. haemolytica, P. multocida, S. typhimurium bacterin toxoid	Texas Vet Lab	Variable (160)
BHV-1 - live vaccine; L. interrogans (Hardjo-Pomona) bacterin	Boehringer Ingelheim Vetmedica	
BHV-1 live vaccine; L. interrogans (Pomona) bacterin	Diamond Animal Health	
BHV-1, BVDV live vaccine; Campylobacter fetus, L. canicola-grippotyphosa-hardjo-icterohaemorrhagiae-pomona bacterin	Zoetis	
BHV-1, BVDV, PIV-3 live vaccine; L. canicola-grippotyphosa-hardjo-icterohaemorrhagiae-pomona bacterin	Colorado Serum Company, Diamond Animal Health	
BHV-1, BVDV, PIV-3, BRSV live vaccine; C. fetus, H. somnus, L. canicola-grippotyphosa-hardjo-icterohaemorrhagiae-pomona bacterin	Elanco US	
BHV-1, BVDV, PIV-3, BRSV live vaccine; C. fetus, L. canicola-grippotyphosa-hardjo-icterohaemorrhagiae-pomona bacterin	Boehringer Ingelheim Vetmedica, Elanco US, Intervet, Zoetis	
BHV-1, BVDV, PIV-3, BRSV live vaccine; H. somnus bacterin	Boehringer Ingelheim Vetmedica	
BHV-1, BVDV, PIV-3, BRSV live vaccine; H. somnus, L. canicola-grippotyphosa-hardjo-icterohaemorrhagiae-pomona bacterin	Boehringer Ingelheim Vetmedica, Elanco US	
BHV-1, BVDV, PIV-3, BRSV live vaccine; L. canicola-grippotyphosa-hardjo-icterohaemorrhagiae-pomona bacterin	Boehringer Ingelheim Vetmedica, Diamond Animal Health, Elanco US, Intervet, Zoetis	
BHV-1, BVDV, PIV-3, BRSV live vaccine; L. canicola-grippotyphosa-hardjo-icterohaemorrhagiae-pomona-M. haemolytica bacterin	Boehringer Ingelheim Vetmedica, Elanco US	
BHV-1, BVDV, PIV-3, BRSV live vaccine; L. hardjo bacterin	Zoetis	
BHV-1, BVDV, PIV-3, BRSV live vaccine; M. haemolytica bacterin	Boehringer Ingelheim Vetmedica, Elanco US	
BHV-1, BVDV, PIV-3, BRSV live vaccine; M. haemolytica toxoid	Boehringer Ingelheim Vetmedica, Zoetis	
BHV-1, BVDV, PIV-3, BRSV live vaccine; M. haemolytica-P. multocida bacterin-toxoid	Diamond Animal Health	
BHV-1, BVDV, PIV-3, BRSV live vaccine; L. pomona bacterin	Diamond Animal Health	
BRV, BCoV killed vaccine; C. perfringens type C, E. coli bacterin-toxoid	Elanco US, Zoetis	Low (162)
BRV, BCoV killed vaccine; C. perfringens type C and D, E. coli bacterin-toxoid	Intervet	
BRV, BCoV killed vaccine; E. coli bacterin	Zoetis	
BVDV live vaccine; C. fetus, L. canicola-grippotyphosa-hardjo-icterohaemorrhagiae-pomona bacterin	Zoetis	Effective (163, 164)
BVDV live vaccine; L. canicola-grippotyphosa-hardjo-icterohaemorrhagiae-pomona bacterin	Zoetis	
BVDV live vaccine; M. haemolytica toxoid	Zoetis	
Trichomonas foetus vaccine, killed protozoa; C. fetus, L. canicola-grippotyphosa-hardjo-icterohaemorrhagiae-pomona bacterin	Boehringer Ingelheim Vetmedica, Elanco US	
C. botulinum type C bacterin-toxoid	United Vaccines	Variable (165)
C. chauvoei-septicum-haemolyticum-novyi-sordellii-perfringens types C and D bacterin-toxoid	Boehringer Ingelheim Vetmedica, Intervet, Zoetis	Low-moderate (166)
C. chauvoei-septicum-haemolyticum-novyi-sordellii-perfringens types C and D, H. somnus bacterin-toxoid	Intervet	
C. chauvoei-septicum-haemolyticum-novyi-sordellii-perfringens types C and D, M. haemolytica bacterin-toxoid	Zoetis	
C. chauvoei-septicum-haemolyticum-novyi-sordellii-tetani-perfringens types C and D bacterin-toxoid	Intervet	
C. chauvoei-septicum-haemolyticum-novyi-tetani-perfringens types C and D bacterin-toxoid	Intervet	
C. chauvoei-septicum-novyi bacterin-toxoid	Colorado Serum Company	
C. chauvoei-septicum-novyi-sordellii bacterin-toxoid	Colorado Serum Company	
C. chauvoei-septicum-novyi-sordellii-perfringens types C and D bacterin-toxoid	Boehringer Ingelheim Vetmedica, Elanco US, Intervet, Zoetis	
C. chauvoei-septicum-novyi-sordellii-perfringens types C and D, H. somnus bacterin-toxoid	Boehringer Ingelheim Vetmedica, Intervet, Zoetis	
C. chauvoei-septicum-novyi-sordellii-perfringens types C and D, M. haemolytica bacterin-toxoid	Zoetis	
C. chauvoei-septicum-novyi-sordellii-perfringens types C and D, Moraxella bovis bacterin-toxoid	Boehringer Ingelheim Vetmedica, Intervet	
C. perfringens type C, E. coli bacterin-toxoid	Elanco US, Intervet, Zoetis	Effective (167)
C. perfringens types C and D bacterin-toxoid	Elanco US, Intervet, Zoetis	
C. perfringens types C and D-tetani bacterin-toxoid	Intervet	
C. tetani-perfringens type D, Corynebacterium pseudotuberculosis bacterin-toxoid	Colorado Serum Company	
C. pseudotuberculosis bacterin-toxoid	Boehringer Ingelheim Vetmedica, Colorado Serum Company	Effective (168)
E. coli bacterin-toxoid	Merial	Variable (169)
H. somnus, M. haemolytica, P. multocida bacterin-toxoid	Texas Vet Lab	Variable
H. somnus, M. haemolytica-P. multocida, S. typhimurium bacterin-toxoid	Texas Vet Lab	Variable
M. haemolytica bacterial extract-toxoid	Elanco US	Moderate (170, 171)
M. haemolytica bacterin-toxoid	Boehringer Ingelheim Vetmedica, Elanco US, Zoetis	
M. haemolytica, P. multocida bacterin-toxoid	American Animal Health, Merial	
P. multocida bacterial extract-M. haemolytica toxoid	Boehringer Ingelheim Vetmedica	Effective (171)
S. typhimurium bacterin-toxoid	Immvac	Low (172)
S. aureus bacterin-toxoid	Hygieia Biological Laboratories	Variable (169)
C. botulinum type B toxoid	Neogen	Variable (165)
C. perfringens type A toxoid	Elanco US, Intervet	Effective (167)
C. perfringens type C toxoid	Colorado Serum Company	
C. perfringens type D toxoid	Colorado Serum Company	
C. perfringens type D-tetanus toxoid	Colorado Serum Company	
C. perfringens types C and D toxoid	Boehringer Ingelheim Vetmedica, Colorado Serum Company	
C. perfringens types C and D-tetanus toxoid	Boehringer Ingelheim Vetmedica, Colorado Serum Company	
Tetanus toxoid	Boehringer Ingelheim Vetmedica, Colorado Serum Company, Intervet, Zoetis, Merck, Santa Cruz Animal Health	Effective (167)

## Antimicrobial and Ancillary Therapies in Cattle

In cattle, the treatment of BRD and scours represents one of the major uses of antimicrobials (173, 174). Because numerous bacteria are involved into BRD development, its treatment normally includes an empirical antimicrobial therapy, including drugs from a wide variety of classes, with penicillins, tetracyclines, macrolides, and quinolones being most frequently used (175). While only a small proportion of animals may show clinical symptoms, treatment is generally applied to the whole herd contributing to improved pathogen control and animal survival (176). However, such an (metaphylactic) approach may result in contamination of the food chain and the environment with antimicrobial resistance (AMR) factors (bacteria and genes) (177). Early detection and treatment of diseased animals allows use of substantially lower doses of antimicrobial drugs, thus reducing the AMR concerns; however, methods and tools for reliable early detection have not been developed (177). Nonetheless in the age of personalized medicine for humans and pets, it is anticipated that such technologies will be forthcoming for livestock as well.

Oxytetracycline and sulfachloropyridiazine administered parenterally and amoxicillin, chlortetracycline, neomycin, oxytetracycline, streptomycin, sulfachloropyridazine, sulfamethazine, and tetracycline administered orally have been approved by the US Food and Drug Administration (FDA) for the treatment of *E. coli* enteritis (colibacillosis) but the evidence of their efficacy is scarce or absent (174). Optimal therapy of cryptosporidial infection in calves remains to be identified with current data suggesting that halofuginone, azithromycin, and lasalocid can be effective (177). The initial observations of increased incidence of diarrhea, reduced growth and malabsorption in healthy calves treated with penicillin, chloramphenicol, and neomycin (174), combined with concerns regarding development of antimicrobial resistance, suggested that use of antimicrobials should be conservative to minimize potential negative effects on animal or human health (178). This and the lack of highly efficient vaccines against most major bovine pathogens has prompted investigations of ancillary and comprehensive strategies to improve and maintain cattle health.

Additional treatments directed at minimizing the effects of pathogens and improvement of non-specific resistance include: optimal nutrition and micronutrient supplementation (nutraceuticals, effects are discussed in the respective section), provision of sufficient amounts of colostrum/milk and feeding it in small doses for optimal digestion of scouring calves, analgesic and anti-inflammatory drugs (including meloxicam and nonsteroidal anti-inflammatory agents) to alleviate intestinal inflammation, pro- and pre-biotics and passive immune therapies.

It has become increasingly evident that a damaged intestine needs nutritional, immune and metabolic/growth factors present in fresh cow’s milk to optimize intestinal repair. Thus, concurrent supplementation of milk and oral rehydration therapy (ORT) solutions resulted in improved intestinal morphology compared to ORT solutions alone (174).

Another emerging approach – aerosol vaccination using BCG vaccine to stimulate memory-like factors of the innate immune system (e.g. ‘trained’ non-specific immunity) was recently evaluated. This immunomodulatory strategy allowed to reduce disease burden in juvenile calves before their adaptive immune system has sufficiently matured (179).

Although not currently recommended as anti-infectious therapies, probiotics can be supplemented (with or without feed) to animals in an attempt to improve performance or increase resistance to enteric pathogens (180). The data on probiotic supplementation in cattle are extremely scarce and somewhat controversial. Lactic acid bacteria or *Lactobacillus rhamnosus* GG supplementation to calves did not decrease *C. parvum* or all cause diarrhea prevalence, respectively (181, 182); whereas *E. coli* Nissle 1917 (for 10-12 after birth) supplementation to calves resulted in a significant decrease in the number of calves developing diarrhea (183).

Chicken egg yolk IgY Abs derived from specific-microbe immunized chickens were efficient in protecting calves against several pathogens including BCoV, RV, CoV, BRSV, enterotoxigenic *E. coli*, salmonella (184–187). Several IgY Ab products are commercially available for calves. Furthermore, a blended treatment using pro-, prebiotics and IgY Abs was successful in reducing all-cause diarrhea in Holstein calves. Supplementation with a combination of *Lactobacillus acidophilus*, *Bacillus subtilis*, *Bifidobacterium thermophilum*, *Enterococcus faecium*, and *Bifidobacterium longum*, prebiotics (RFC-MOS, FOS), charcoal, and dried egg protein from hens hyperimmunized with K99+ *E. coli* antigen, *S. enterica* var. Typhimurium and Dublin, CoV, and RV resulted in a decreased incidence of diarrhea during the first 3 weeks of life of calves (25.0% *vs*. 51.1% in the control group) (188).

Additional therapies have included maintenance of low abomasal pH to reduce numbers of pathogenic bacteria (such as salmonella), supplementation of short-chain fatty acids (acetate and propionate) to inhibit growth of pathogenic bacteria, homeopathic treatments (such as podophyllum or oregano), use of intestinal ‘protectants’ and ‘absorbants’ (such as kaolin, activated attapulgite, pectin, and activated charcoal), administration of agents that decrease intestinal motility, such as hyoscine N-butylbromide or atropine. However, clinical efficacy was very limited and needs further evaluation (174). Similarly, parenteral administration of mycobacterial cell wall extracts (189) had some efficacy in treatment of calf diarrhea, but wide-scale confirmatory evaluation studies have not been conducted.

Thus, there appears to be no broadly applicable therapeutic treatments (with the exception IgY, ORT and antibiotics) that could drastically improve health of dairy cattle. The most practical approach should likely rely on a combination therapy utilizing passive immune protection, vaccination and supplemental treatments using probiotics/probiotic products.

## Concluding Remarks

The insufficient basic knowledge of bovine immune function and the lack of a developed toolbox for immunological studies hinder our ability to generate and maintain optimal cattle health. This is especially challenging for newborn dairy calves (due to immune immaturity) and dairy cows during the transition period (due to metabolic and immune dysfunction). Further evaluation and optimization of the existing vaccines in large-scale field trials are critical. In-depth studies of the influence of various macro- and micronutrients and commensal and probiotic bacteria on bovine immune function will likely yield novel and urgently needed interventions to combat infectious diseases and inflammatory disorders in cattle. The existing evidence suggests that provision of cow colostrum and milk in sufficient amounts in the first week of life and optimization of cattle feeding according to the production stage and group promote immune development and help to maintain immune function and should be widely adopted. Additionally, emerging evidence suggest that a second colostrum feeding soon after the first one may provide appreciable health benefits. Routine immunizations of calves, heifers and pregnant cows against vaccine-preventable diseases and elimination of the pathogens that subvert or compromise the immune system and are essential strategies that can help to maintain a healthy herd. Further, conducting additional research to determine optimal timing for vaccination and application of novel vaccines and adjuvants (micronutrients, probiotics, etc) will allow improved vaccines and vaccination protocols (such as vaccination of recently transported stressed cattle) to achieve better protection by new or existing vaccines. Finally, a combination of available ancillary strategies discussed above, including pro- and prebiotic feeding, IgY Ab supplementation, controlling abomasal pH and preserving epithelial integrity as well as minimizing the use of antimicrobials are expected to have a positive impact on dairy production.

## Author Contributions

AV generated the initial draft. AV and LS have revised the final content critically. All authors contributed to the article and approved the submitted version.

## Conflict of Interest

The authors declare that the research was conducted in the absence of any commercial or financial relationships that could be construed as a potential conflict of interest.
